# Phone-based motivational interviewing to increase self-efficacy in individuals with phenylketonuria

**DOI:** 10.1016/j.ymgmr.2016.01.002

**Published:** 2016-01-18

**Authors:** Krista S. Viau, Jessica L. Jones, Maureen A. Murtaugh, Lisa H. Gren, Joseph B. Stanford, Deborah A. Bilder

**Affiliations:** a Department of Pediatrics, University of Utah, 295 Chipeta Way, Salt Lake City, UT 84108, USA; b Department of Family and Preventive Medicine, University of Utah, 375 Chipeta Way, Suite A, Salt Lake City, UT 84108, USA; c Department of Internal Medicine, University of Utah, 295 Chipeta Way, Salt Lake City, UT 84108, USA; d Department of Psychiatry, University of Utah, 650 Komas Drive, Suite 200, Salt Lake City, UT 84108, USA

**Keywords:** IEP, individualized education program, ID, intellectual disability, IQR, interquartile range, MI, motivational interviewing, PAM, Patient Activation Measure, Phe, phenylalanine, PKU, phenylketonuria, SOC, stages of change, Phenylketonuria, Motivational interviewing, Self-efficacy, Stages of change

## Abstract

**Objective:**

To measure change in patient activation and self-efficacy in individuals with phenylketonuria (PKU) before and after a 6-month phone-based motivational interviewing (MI) intervention and determine the feasibility of implementing dietary counseling for PKU using an MI approach.

**Methods:**

Participants (*n* = 31) included preadolescents (7–12 years), adolescents (13–17 years), and adults (18–35 years) with early-treated PKU. Participants completed online questionnaires assessing self-reported stage of change (SOC), patient activation, and self-efficacy for PKU self-management behaviors. The intervention included monthly phone-based dietary counseling using MI during which participants set monthly goals.

**Results:**

Patient activation and self-efficacy were significantly different by age group (both *p* < 0.01) with higher scores in older participants. Self-efficacy significantly increased from baseline to month 6 among adolescents and adults (7.4 ± 1.9 and 8.6 ± 1.3, respectively, *p* = 0.002). Preadolescents did not have a significant change in self-efficacy (*p* = 0.79). There was no increase in patient activation for preadolescents or adolescents/adults (*p* = 0.19 and *p* = 0.24, respectively). Indicators of learning problems were not significantly associated with self-efficacy (*p* = 0.33) or patient activation (*p* = 0.83).

**Conclusion:**

These results demonstrate the feasibility of implementing phone-based dietary counseling for PKU using MI. This study also supports further investigation of MI as an intervention approach to improving self-efficacy and self-management behaviors in adolescents and adults with PKU.

## 1 Introduction

Poor treatment adherence is frequently reported among individuals experiencing chronic illness [1], [2], [3], [4], including those with phenylketonuria (PKU). In individuals with PKU, there are multiple studies reporting rising blood phenylalanine (Phe) concentrations with age [5], [6], [7] and high rates of loss to follow up with only an estimated 23% of adults with PKU receiving treatment from a metabolic center in 2012 [8], [9].

Adherence to daily medical and/or dietary regimens becomes increasingly more difficult as treatment complexity increases [10], [11]. The ability to adhere to PKU treatment recommendations may be further impaired by subtle cognitive deficits or psychiatric symptoms [12], [13]. Treatment of PKU is multifaceted, and successful management includes daily monitoring of dietary Phe or protein intake, consumption of a Phe-free medical formula, weekly to monthly monitoring of blood Phe levels, and regular food records and appointments with a metabolic clinic [14]. In a study of 19 families with PKU, all children reported difficulty following the diet and 91% reported problems drinking medical formula. Maladaptive strategies to address these difficulties were correlated with elevated blood Phe levels [15]. Strategies to facilitate behavior change are needed to improve treatment adherence in individuals with PKU.

Tailored interventions based on an individual's self-reported readiness to change have been shown to be more effective for improving health behavior change compared to more traditional approaches [16], [17], [18], [19], [20]. Stages of change (SOC), first described by James Prochaska and Carlo DiClemente [21], are a means of conceptualizing the process people experience as they evolve from having minimal insight and/or desire to change a behavior to the point at which they have changed this behavior and maintained this change. SOC reflect one's readiness to engage in behavior change [21], [22]. Motivational interviewing (MI) is an intervention that incorporates a provider's understanding of their patient's current SOC to provide tailored counseling to support the patient's ability to progress towards the desired behavioral change. MI is a patient-centered, collaborative style of communication that elicits a patient's intrinsic motivation for change. MI employs strategies, such as reflective listening, supporting patient autonomy to decide whether or not to change, monitoring the patient's degree of readiness to change, affirming the patient's choices, and reducing resistance. The practitioner guides the patient and helps them identify discrepancies between their current actions and desired outcomes, creating opportunity for the patient to develop motivation to change [23], [24], [25], [26], [27].

In several chronic medical conditions, multiple studies and meta-analyses support the effectiveness of MI for improving self-efficacy, self-management behaviors, and health outcomes [28], [29], [30], [31], [32], [33]. For example, one meta-analysis reported improved self-efficacy with MI in patients with diabetes, cardiovascular disease, or smoking, producing an overall effect size of 1.39 (95% CI 1.09-1.78) [31]. MI has been associated with subjective and objective measures of improved disease management including self-monitoring (blood sugar, food intake, and exercise), glycemic control, blood pressure, cholesterol, HIV viral load, and body weight [31], [34], [35]. MI has also demonstrated positive results in a variety of settings [28], [36], [37], [38], and age groups [32], [33], [39], [40].

Patient activation also appears to mediate health behavior change. Patient activation refers to the knowledge, skills, confidence, and motivation to manage one's own health [41]. Increased patient activation has been consistently associated with improved self-management behaviors, health outcomes, and reduced health care costs among individuals with a variety of chronic diseases [11], [19], [42], [43], [44], [45], [46], [47], [48]. Improvements in patient activation occur over a continuum and can be measured across self-care skills rather than linked to a single targeted behavior [49]. This characteristic lends itself to studying patient progress towards better disease self-management across multiple health behaviors related to this goal.

Like patient activation, increased self-efficacy coincides with improved health behaviors. Self-efficacy is the belief that one can successfully implement a behavior required to achieve the desired outcome [22]. Meta-analyses have reported higher self-efficacy is associated with increased participation in recommended self-care in adults with type 1 and 2 diabetes and adherence to antiretroviral therapy in individuals with HIV [11], [48]. Self-efficacy appeared to be a critical component in moving from goal setting to goal completion in a study of adolescent females making healthy food choices [47]. Crone et al. found that increased parental self-efficacy regarding medical formula intake (*p* = 0.007) and raising a child with PKU (*p* = 0.028) was associated with improved blood Phe levels in their children [50]. Enhancing self-efficacy among individuals with PKU, as with other chronic diseases, may serve an important role in increasing treatment adherence related to PKU.

The aim of the current before-and-after study was to (1) measure change in patient activation and self-efficacy during the course of the intervention and (2) determine the feasibility of implementing dietary counseling for PKU using an MI approach. As a frontier state, Utah's population is spread over 85,000 mile^2^. The catchment area of Utah's primary PKU clinic includes Utah, Idaho, and Wyoming. Because many patients residing in other states also face challenges related to the physical proximity of their closest PKU clinic, an additional component of this study was the implementation of intervention through phone contact, rather than in-person.

## 2 Methods

### 2.1 Participants

Study participants were individuals aged 7–35 years, diagnosed with PKU on newborn screening and treated within one month of birth, English speaking and had internet access at home. Exclusion criteria included 1) intellectual disability (IQ < 70), 2) pregnancy, as excess Phe is teratogenic and requires different treatment guidelines [51], 3) hyperphenylalaninemia not requiring dietary treatment, and 4) concurrent participation in clinical trial(s) testing enzyme substitution therapy. Patients were identified and recruited through the Utah Metabolic Clinic from December 2013 to July 2014. Participants were compensated monetarily for their time. The University of Utah Institutional Review Board approved this study and written consent – and child assent, if appropriate – was obtained for all participants.

### 2.2 Measures

#### 2.2.1 Stages of change

A questionnaire based on a format previously shown to be reliable was compiled to assess participants' current SOC [52], [53]. Treatment of PKU is complex and multiple behaviors contribute to blood Phe levels [54]. The following three behavioral domains were chosen to assess SOC: meeting dietary Phe/protein goals, reaching medical formula goals, and making healthy food choices. Although, healthy food choices may not affect Phe levels to the extent that Phe/protein and medical formula do, the inclusion of this domain provided opportunity to discuss other areas of improvement for those already meeting Phe/protein and formula goals. This goal also provided an option for participants not yet ready to discuss working towards Phe/protein or formula goals. Participants were asked to select the most important behavior from a list within each behavioral domain (Table 1). The participant could write in an additional behavior of interest if items listed were not felt to be pertinent. For each domain, the SOC was scored on a progressive scale from 1 (*absence of the desire to change behavior*, *precontemplation*) to 5 (*presence of the desire to maintain a changed behavior*, *maintenance*).

#### 2.2.2 Patient activation

Activation was measured with the PAM-13. This questionnaire measures perceived knowledge, ability, and confidence to manage one's health [49]. PAM-13 scores range from 0 to 100, and this score can then be divided into four levels of activation [41]. These levels reflect a patient's belief that he/she should play an active role in self-care and collaborate with providers (level 1), knowledge about one's disease and its treatment (level 2), confidence to support new behaviors (level 3), and ability to maintain lifestyle changes in times of stress (level 4) [49]. The PAM-13 has been validated in adults, but not in children [49].

#### 2.2.3 Self-efficacy

Self-efficacy was measured with a modified version of the 8-item Diabetes Self-Efficacy Scale developed at the Stanford Patient Education Research Center [55], [56]. Items were ranked on a 10-point Likert scale ranging from 1 (*not confident*) to 10 (*totally confident*). The total score reflected the average of the eight items rather than the sum to maintain the original metric. The wording of items 2, 5, 6, and 8 was revised to be applicable to PKU (e.g., substituting “blood sugar” with “phenylalanine”). Similarly, the topics of item 1 and 4 were replaced with statements regarding the ability to consume medical formula and collect blood samples, as the original topics (frequent meals and exercise) were not components of PKU management.

#### 2.2.4 Blood phenylalanine

Participants collected a blood Phe sample via finger stick at enrollment and were asked to collect monthly blood samples as part of standard of care monitoring during the study.

### 2.3 Intervention

Summarizing and goal setting are important elements of MI. The intervention included three components of MI: summary report, monthly phone-based dietary counseling, and goal setting.

#### 2.3.1 Summary report

A monthly PDF was emailed to participants to summarize previous responses. It displayed each participant's current SOC for selected behaviors within the three domains, monthly goal, prescribed protein intake, and average protein intake over the past month estimated from a validated food frequency questionnaire [57]. Participants received the summary prior to phone-based MI with instructions to review the information prior to the call, though they were not required to view the summary during the conversation.

#### 2.3.2 Phone-based motivational interviewing

Participants were contacted by phone once per month during the 6-month intervention. Author KV delivered the MI intervention. The interventionist received more than 30 h of MI training via workshops, individual coaching and expert practice feedback. The sessions were audio recorded and a random sample was reviewed by contributor MA, who was also trained in MI, to evaluate reliability of the MI intervention and give practice feedback. Calls were made after metabolic clinic staff received monthly blood Phe results, generally 5–10 days after sample collection. For participants < 18 years of age, telephone counseling was conducted with either (1) the caregiver and participant concurrently or (2) the participant alone followed by a verbal summary provided to the caregiver.

The interventionist reviewed topics on the summary and the participant's blood Phe result at the beginning of the session. After the initial discussion, participants were asked if he/she would like to focus on anything in particular the following month. A list of options was presented if the participant did not have a particular topic in mind. MI techniques aimed to understand the participant's goals and values, barriers to change, and elicit personal motivation for change were used during the session to explore potential behaviors to target and, if appropriate, to create a monthly goal.

#### 2.3.3 Monthly goals

Topics for monthly goals were derived from the discussion, particularly participant change talk, in which the participants' words favored behavior change. While current SOC for behavioral domains were discussed during the phone conversation, the participant had the option of choosing a goal that was separate from the behaviors selected on the SOC questionnaire. Each month participants were asked if they would like to form a goal, though they were reminded they could choose to maintain current health if they did not identify a specific change they were ready to implement. If the participant was interested in creating a goal, the interventionist assisted to design goals that met SMART guidelines [58]. The interventionist reviewed the participant-reported progress of the previous month's goal and discussed next steps during the following month's telephone conversation.

### 2.4 Data collection

Study data were collected and managed using the REDCap electronic data capture tools hosted at University of Utah [59]. Participants were instructed on questionnaire completion during an in-person baseline visit. All questionnaires were emailed to participants and completed online using REDCap survey tools. Participants under 18 years of age were asked to complete the questionnaires with a caregiver. KV clarified individual responses with participants over the phone as needed. The SOC and food frequency questionnaires were administered monthly. Patient activation and self-efficacy questionnaires were completed at baseline and months 3 and 6.

Demographic and treatment information including date of birth, sex, current protein and medical formula prescriptions, and eligibility for an individualized education program (IEP) were obtained from the electronic medical record. IEP eligibility was used as a proxy for the presence of learning problems. Adult participants were classified based on previous IEP eligibility.

### 2.5 Data analysis

Demographic, questionnaire and monthly goal data were summarized using descriptive statistics: continuous variables were reported as mean ± standard deviation if normally distributed and median (interquartile range) if not normally distributed, and categorical variables were reported as frequencies. Differences between data at baseline were assessed via Fisher's exact test for categorical data and one-way ANOVA for continuous data. Correlation between total goals created and total goals achieved was assessed with Spearman correlation coefficients due to the small sample size.

Due to repeated measurements in each participant, we used random effects linear regression to evaluate differences between IEP eligibility, age categories, and number of goals created and achieved. Random effects linear regression was also used to compare baseline patient activation and self-efficacy to month 3 and month 6 scores, controlling for potential confounders. We used an intention-to-treat analysis. Statistical analyses were performed using Stata 13.0 (StataCorp, College Station, TX). Significance was defined as a two-sided *p*-value with alpha of 0.05.

## 3 Results

**Fig. 1:**
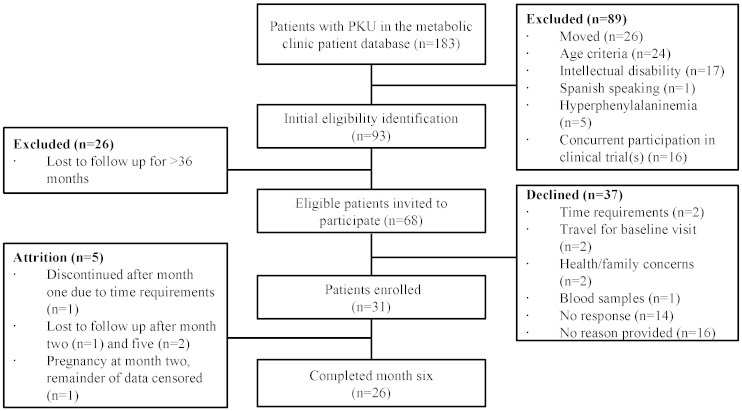
Flow chart of participant recruitment, enrollment, and retention. Patients in the metabolic clinic database include all known patients in Utah who have been seen in our clinic and a portion of patients from surrounding states, including Idaho and Wyoming.

Forty-six percent of patients invited (*n* = 31) to participate enrolled in the study (Fig. 1). Participants fell into three age categories, preadolescents (7–12 years), adolescents (13–17 years), and adults (18–35 years, Table 2). Approximately half (*n* = 17) of participants' blood Phe concentrations were above the therapeutic range at enrollment (120–360 μmol/L) [51]. Participants completed a median (IQR) of 5 (4–6) of the 6 monthly phone-based MI sessions. The sessions were brief with the median (IQR) duration of 13 min (10.5–16). Thirty-one participants completed baseline questionnaires, 28 completed month 3 questionnaires, and 26 completed month 6 questionnaires.

### 3.1 Stages of change

At baseline, most participants reported being in action or maintenance stages for PKU management behaviors (Table 3), and no participants were pre-contemplative for all three behavioral domains. There was no significant difference in baseline SOC between age categories or baseline Phe category (> or ≤ 360 μmol/L) for any domain (data not shown). Participants indicated their current SOC for one behavior per month within each domain. Most chose different behaviors throughout the intervention (*n* = 20 for Phe/protein goals, *n* = 13 for formula goals, and *n* = 20 for healthy choices). The number of different behaviors selected for the three behavioral domains were not significantly associated with the SOC for that domain (data not shown). Of the seven participants who were pre-contemplative or contemplative at baseline, three did not complete month 6 measures while four completed the study and reported increasing SOC scores during the study's course. Because all participants were not consistent across time points in regards to the behaviors on which they chose to focus, hypothesis testing was not performed for change in SOC across the intervention.

### 3.2 Monthly goals

Participants created a total of 118 goals during 150 cumulative counseling sessions. Goals varied throughout the intervention with a median (IQR) of 3 (2–4) distinct goals per participant. Of these, 58.4% of goals were achieved per participant report. The number of different goals was not correlated with the percentage of goals achieved (*p* = 0.80). Monthly goals were not always consistent with the behaviors selected on the SOC questionnaire. Nearly one third (31.4%) of participant goals were focused on Phe/protein intake, 22% medical formula intake, 21.2% healthy choices, 17.8% exercise, 5.1% regular medication intake, and 2.5% non-health related topics. The total number of goals created and the number of goals achieved during the intervention were not significantly different by IEP eligibility (*p* = 0.36 and *p* = 0.59, respectively) or associated with baseline stage of change (*p* = 0.63 and *p* = 0.63, respectively), patient activation (*p* = 0.66 and *p* = 0.45, respectively), or self-efficacy scores (*p* = 0.93 and *p* = 0.99, respectively).

### 3.3 Patient activation and self-efficacy

At baseline, most participants had PAM scores reflecting high levels of activation (13% level 3 and 58% level 4), and moderate self-efficacy scores (Table 4). Participants who completed the intervention and those who discontinued prior to 6 months (Fig. 1) had similar median (IQR) patient activation scores (71 (53–78) and 69 (50–73), *p* = 0.94) and self-efficacy scores (7.9 (5.4–8.8) and 7.3 (5.1–8.8), *p* = 0.64) at baseline. Over the course of the study, 4 participants maintained their baseline activation scores, 15 participants increased activation scores, and 4 participants decreased activation scores. Total self-efficacy scores increased for 19 participants during the intervention and decreased for 7 participants. There was no significant difference in the change in PAM or self-efficacy scores over the 6-month intervention by baseline Phe category (*p* = 0.52 and *p* = 0.17, respectively).

Self-management responsibilities increase with age, [60] and a concomitant increase in activation and self-efficacy were expected. In our participants, patient activation and self-efficacy scores were significantly different among age categories (*p* < 0.01 for both). The mean activation score was 61.4 ± 18.5 for preadolescents, 70.5 ± 18.9 for adolescents, and 74.5 ± 14.6 for adults. The mean self-efficacy score was 6.7 ± 1.7 for preadolescents, 7.7 ± 1.5 for adolescents, and 8.1 ± 1.7 for adults.

**Fig. 2:**
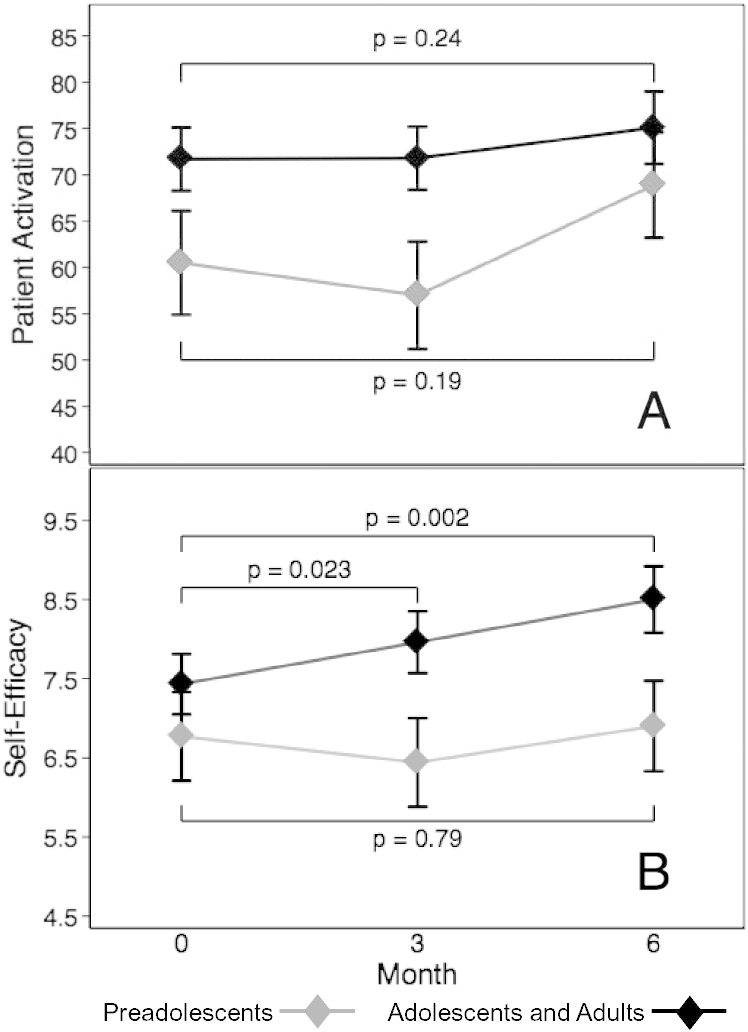
Predicted means ± standard error for patient activation (A) and self-efficacy (B) for preadolescents and adolescents/adults from baseline to month 3 and month 6. Random effects linear regression used to estimate slope in patients with PKU. Patient activation was scored from 0 to 100, and self-efficacy was scored from 1 to 10. Higher scores reflected increased activation or self-efficacy for both measures.

The change in patient activation and self-efficacy was assessed with random effects linear regression (Fig. 2). IEP eligibility was not included in the model, as it was not significantly associated with patient activation or self-efficacy in bivariate regression models (*p* = 0.83 and *p* = 0.33, respectively). Considering the difference in mean activation and self-efficacy among age groups, we stratified the analysis by age category. However, adolescents and adults were combined into a single category, as there were only five participants in the adolescent category. Patient activation did not significantly change over the course of the intervention for either group (Fig. 2A). However, self-efficacy scores significantly increased among adolescents and adults from baseline to month 3 (*β* = 0.52, *p* = 0.023) and month 6 (*β* = 1.07, *p* = 0.002), though this was not found in the preadolescent group at month 3 or 6 (*p* = 0.38 and *p* = 0.79, respectively; Fig. 2B).

## 4 Discussion

This study describes the implementation of phone-based dietary counseling using motivational interviewing to support patients' efforts at managing PKU. Patients' knowledge and engagement in their own care are crucial for chronic disease management. Incorporating motivational interviewing into dietary counseling for PKU provides an opportunity to enhance motivation while teaching skill building in this patient population.

Measures of self-efficacy reflect participants' beliefs that they can successfully implement a behavior required to achieve the desired outcome. At the start of this study, participants reported a moderate degree of self-efficacy in regards managing their PKU. After the 6-month MI intervention, self-efficacy significantly increased (Fig. 2B) among adolescents and adults demonstrating an improvement in self-confidence for managing a variety of behaviors related to PKU treatment. However, increase in self-efficacy was not found among preadolescents. This may be related to the variation in self-management skills expected at different ages. Adolescents and adults have greater self-management responsibilities compared to younger participants; therefore, confidence regarding some PKU management skills may be applicable to older participants.

MI has been demonstrated to be beneficial with patients of a variety of ages [31], [33]; yet, developmental stages and unique PKU-related stressors merit special consideration when evaluating MI in this population. This sample included participants aged 7–35 years. While it is crucial to engage younger patients and provide opportunity for skills mastery [54], [61], [62], differences in the efficacy of MI in the youngest participants compared to adult participants were identified. This finding may be attributed to inherent limitations of early developmental stages in regards to understanding illness-related concepts, expressing thoughts and feelings verbally, and linking current behavior with past events or long-term goals [63]. Therefore, motivational interviewing targeting preadolescents with PKU would be most effective when engaging both parents and children in the intervention process and identifying common goals to facilitate their implementation [32]. In contrast, MI is well suited for adolescence considering this period is typically characterized by ambivalence, desire for autonomy, and development of self-identity [63], [64], [65]. Additionally, MI may be a beneficial component to initiatives that engage adult patients lost to follow up in a metabolic clinic [8].

Participants' SOC assessment prior to the monthly MI intervention provided the context of their current readiness to take action and a starting point for therapeutic discussion. Most participants changed their selected SOC behaviors over the study's 6-month duration (Table 1). The exploratory nature of MI may encourage participants to select a behavior that they had not considered previously. While demonstrating the flexibility of the intervention, this variability limited the use of SOC as an outcome variable. Subsequently, the degree to which behavior-specific SOC change reflected a lack of readiness for the behavior change versus the successful implementation of the behavior remains unclear. During future studies, SOC could be collected for a variety of specified self-management behaviors to clarify how MI may be associated with readiness to change for specific behaviors over time in a PKU population.

Subtle intellectual and/or executive deficits may impair one's ability to manage the complex treatment for PKU. Studies in other populations have reported that executive functioning impairment was associated with poor self-management and/or treatment adherence [66], [67]. In contrast, our results did not identify a significant difference in the number of goals created or achieved, patient activation, or self-efficacy scores between individuals with versus without learning problems. MI was developed for and has demonstrated efficacy in the treatment of substance abuse [39], [68], [69], which itself is associated with elevated rates of mental illness and executive deficits [70]. MI has also been successfully used to increase engagement in patients with traumatic brain injury [71]. Although the current study excluded individuals with intellectual disability, the absence of a notable correlation between the presence of learning problems and outcome measures support further exploration of MI as a potential intervention to facilitate treatment adherence in individuals with PKU and consideration of extending future study populations to include individuals with more significant intellectual deficits.

### 4.1 Limitations

This study was designed to explore the feasibility of implementing an MI dietary intervention for individuals with PKU residing across a substantial geographical area and to assess its potential to demonstrate positive health behavior outcomes. The study design introduced potential for selection and self-report bias. These results cannot be generalized to adults with PKU who no longer seek care at metabolic clinics or who would decline participation in a dietary-based intervention study. Characteristics of these adults may differ from those of study participants in several regards including baseline SOC, activation, self-efficacy, and metabolic control. Additionally, in the absence of a control group, the degree to which the Hawthorne effect contributed to improvement in outcomes measures is unknown. Although IEP eligibility served as a proxy for the presence of learning impairment, not all students with learning difficulties receive an IEP; conversely, students with an IEP may be eligible as a result of non-cognitive issues that affect their ability to learn such as behavioral or emotional disturbance. As a rare disease, PKU intervention studies are inherently limited by the challenge of recruiting sufficient numbers of participants to provide adequate power.

### 4.2 Conclusion

Phone-based dietary counseling using motivational interviewing was associated with a significant increase in self-efficacy for self-management behaviors among adolescents and adults with PKU following six months of intervention. This change was not accompanied by improvements in patient activation. The presence of learning problems was not associated with health behavior outcome measures. These results demonstrate the feasibility of implementing phone-based dietary counseling using MI in a PKU population and support further investigation of MI as an intervention approach to improving self-efficacy and self-management behaviors in adolescents and adults with PKU, particularly for patients living in remote locations.

## Figures and Tables

**Table 1:** List of behavioral targets for stage of change questionnaire.

Domain	List of behaviors
Dietary Phe/protein	Count how much Phe/protein I eat Keep diet records Plan meals beforehand Watch my portion sizes Prepare meals at home
Medical formula	Drink all formula every day Drink formula several times per day Make my own formula Bring my formula to school/work Try a different formula or try to improve the taste of my current formula
Healthy food choices	Eat more fruits/vegetables Drink fewer sweetened drinks (soda, juice) Eat out less often (restaurants, fast food) Eat fewer “junk foods” (chips, cookies, candy) Cook meals at home more often

Phe, phenylalanine.

*Note*: Participants were asked to “Choose the single most important thing you could personally do to meet your goals” for each domain.

**Table 2:** Demographic and baseline treatment information for participants with PKU presented as median (IQR) or frequency.

	Total *n* = 31	Preadolescent (7–12 years) *n* = 11	Adolescent (13–17 years) *n* = 5	Adult (≥ 18 years) *n* = 15	*p*-Value^c^
Age (years)	17.2 (9.8–26.5)	9.5 (7.5–11.5)	16.4 (15.1–16.8)	26.5 (20.4–30.2)	
Phe level at enrollment (μM)	390 (209–579)	317 (140–433)	573 (495–865)	491 (226–686)	0.036
Female	22	8	1	13	0.032
Overweight/obese	16	2	3	11	0.016
IEP^a^	7	2	1	4	1.00
Taking medical food^b^	28	10	5	13	1.00
BH4 treatment	14	8	1	5	0.09

BH4, tetrahydrobiopterin; IEP, individualized education program; IQR, interquartile range; Phe, phenylalanine.

a Participants currently or previously eligible for an IEP in school as an indicator of learning impairment.

b Participants reporting regular medical food intake at enrollment.

c Differences between groups assessed with Fisher's exact test for categorical data and one-way ANOVA for continuous data.

**Table 3:** Baseline stage of change for three behavioral domains.

	Phe/protein goals	Formula goals	Healthy food choices
Precontemplation	1	–	–
Contemplation	6	–	5
Preparation	5	5	5
Action	5	6	10
Maintenance	14	20	11

*Note*: Baseline stage of change reflects a behavior with each domain selected by each participant.

**Table 4:** Individual self-efficacy scores by question for participants with phenylketonuria presented as mean ± SD where 1 = not confident and 10 = totally confident.

Questionnaire item	Baseline (*n* = 31)	Month 3 (*n* = 28)	Month 6 (*n* = 26)	Change in mean scores
You can do all the things necessary to manage your PKU on a regular basis.	7.0 ± 2.2	7.5 ± 2.1	8.0 ± 2.3	+ 1.0
You can follow your diet when you have to prepare or share food with other people who do not have PKU.	7.5 ± 2.5	7.5 ± 2.0	8.5 ± 1.4	+ 1.0
You can choose the appropriate foods to eat when you are hungry (for example, snacks).	7.5 ± 2.4	7.9 ± 1.4	7.9 ± 1.8	+ 0.4
You can poke your finger to collect a blood sample every month at a minimum.	7.7 ± 2.9	8.0 ± 2.8	7.8 ± 2.7	+ 0.1
You can do something to prevent your blood phenylalanine levels from increasing.	6.7 ± 2.4	7.1 ± 2.4	7.5 ± 2.5	+ 0.7
You know what to do when your blood phenylalanine level goes lower or higher than it should be.	7.1 ± 2.8	6.9 ± 2.4	7.7 ± 2.6	+ 0.6
You can judge when changes in your illness mean you should visit the doctor.	6.3 ± 2.7	6.3 ± 2.8	7.1 ± 2.7	+ 0.8
You can control your PKU so that it does not interfere with the things you want to do.	7.9 ± 2.3	8.0 ± 1.7	8.6 ± 1.6	+ 0.7

*Note*: Questions from modified version of the Stanford Diabetes Self-Efficacy Scale [56].
